# Advantages and limitations of implant surgery with CAD/CAM surgical guides: A literature review

**DOI:** 10.4317/jced.55871

**Published:** 2020-04-01

**Authors:** Gokce-Soganci Unsal, Ilser Turkyilmaz, Samantha Lakhia

**Affiliations:** 1 Assistant Professor, Department of Prosthodontics, Faculty of Dentistry, Yildirim Beyazit University, Ankara, Turkey; 2 Clinical Associate Professor, New York University College of Dentistry, Department of Prosthodontics, New York, NY, USA; 3 Third-year Dental Student, New York University College of Dentistry, New York, NY, USA

## Abstract

**Background:**

The purpose of this study is to review the available literature associated with implant surgery using computer-aided design/computer-aided manufacturing (CAD/CAM) surgical guides and discuss the advantages and disadvantages of this advanced technique.

**Material and Methods:**

An electronic literature search was conducted in the PubMed database for the relevant information on implant placement with CAD/CAM surgical guides. This review was constructed following PRISMA (Preferred Reporting Items for Systematic Reviews and Meta-Analyses) guidelines. Articles were limited to those published within the past 10 years and in the English language. Only clinical studies were included. Inclusion criteria were: studies including 10 implants or more and studies presenting angular deviations in degrees and linear deviations in millimeter. Observational studies, reviews, animal studies, in vitro studies, case reports, simulation studies were excluded. Nine articles were included for qualitative synthesis.

**Results:**

The initial search detected 61 articles, and after screening abstracts, a total of 15 articles were selected for full-text review. After the full-text analysis of the 15 articles, six articles were excluded as they did not meet inclusion criteria for study design, study population, and implant placement with data presentation for angular and linear deviations. Ultimately, nine articles providing angular and linear deviations between planned and actual placed implants were used in this review. Common problems that may be encountered by clinicians were listed, and recommendations were made on how to avoid those problems.

**Conclusions:**

It has been suggested that although unrealistic expectations are often associated with implant placement with CAD/CAM surgical guides, there is no impeccable accuracy in the clinic. This review demonstrated that the practitioners should be aware of the angular and linear deviations up to 5 ° and 2.3 mm. Therefore, inexperienced dentists should obtain adequate training and be familiar with the basic steps with CAD/CAM surgical guides to avoid complications.

** Key words:**CAD/CAM, CBCT, implant, stereolithography, surgical guide.

## Introduction

The loss of just one tooth will eventually have a global impact on the entire stomatognathic system. Bone loss, shifting of teeth, occlusal changes, decreased masticatory force and many more effects are felt throughout the entire system (1,2). In order to prevent the progression of these effects, dentistry has continually searched for the ideal tooth replacement. With the advent of dental implants, clinicians can now restore patients higher levels of health and function than ever before (3,4).

When a patient presents with a need for implants to replace missing teeth, correct execution can only occur with thorough planning (5,6). When restoring with dental implants, the implants can be placed in an ideal, predicTable, and planned location by using recently introduced technology such as cone-beam computed-tomography (CBCT), 3-dimensional (3-D) implant planning software, and surgical guide utilizing computer-aided design and computer-aided manufacturing (CAD/CAM) (7-9). Implant dentistry is rapidly evolving and constantly challenging the practitioner to be aware of recent advances. Though it may feel overwhelming for a practitioner to stay informed with the continuous introduction of new technologies (4,6).

CBCT does not only provide valuable information for evaluation before placing dental implants but it also translates into completely digital planning of surgical cases (10). Utilizing a CBCT scan, a CAD/CAM surgical guide may be fabricated based on the precise location of a planned implant (11). All of the major implant companies offer software which can be used for planning the specific location of implants in the CBCT image, and eventually a surgical guide can be fabricated.

The purpose of using virtual implant software is to plan the placement of the implants in prosthodontically driven positions (12). Of course an implant may be placed anywhere the bony anatomy allows, but in order to build a successful prosthesis for that implant, the correct planning must be done. The benefits of virtual planning and fabricating CAD/CAM surgical guides from the planning are numerous (13-15). The patient’s chair time is decreased, the surgery is more predicTable and less stressful, the implants are placed in a restoratively driven manner, and the case difficulty is learned ahead of time (13-15). On the other hand, there are some limiting factors involving CAD/CAM surgical guides such as sufficient mouth opening, which must be evaluated before ordering the guide. The patient must have adequate opening depending on the guide, length of implant, and drill system used. Otherwise, even if the surgical guide is accurately fabricated, it may not be inserted in the mouth properly and eventually the surgery can not be performed.

Three different surgical guide designs depending on their supporting surfaces have been described (16); a) tooth-supported surgical guide is placed on the remaining natural teeth; b) mucosa-supported surgical guide is directly placed on the mucosa, allowing flapless implant placement; c) bone-supported surgical guide is placed on the bone following a full-thickness mucoperiostal ﬂap elevation.

Implant placement with CAD/CAM surgical guides is a highly technique sensitive procedure. All steps from virtual planning to the actual surgical procedure need to be executed diligently as it comprises of a serial of diagnostic and therapeutic events (17,18). Generally, computer-guided surgery workflow includes seven steps in order; clinical diagnostics, diagnostic tooth setup (interim denture), fabrication of radiographic guide, digitization with CBCT scan, 3-D diagnostics and treatment planning, fabrication of surgical guide, surgical operation. The total amount of placement inaccuracy is determined by the sum of mistakes that occur during those steps (19,20). There is a learning curve for this method and the transition from the mechanical workflow to the digital workflow may be difficult for some clinicians (19,20). Hence, clinicians should obtain comprehensive training prior to getting involved in similar types of advanced treatments. It is important to note that there is no perfect computer-guided surgical method at this moment. Because, several studies have already indicated some degrees of angular, vertical and horizontal deviations from the digital plan to the actual surgery (21-35). It should be kept in mind that greater deviations and major complications such as nerve injury may be caused by untrained and novice clinicians (30,31).

The purpose of this study is to review the dental literature regarding implant placement with CAD/CAM surgical guides, and emphasize advantages and limitations of this recently popular method.

## Material and Methods

In April of 2019 a literature review was conducted in the Medline, PubMed database following PRISMA (Preferred Reporting Items for Systematic Reviews and Meta-Analyses) guidelines.

-Search Strategy.

The search terms were: “CAD/CAM”, “surgical guides” and “dental implant”. A search was conducted utilizing Boolean operators “AND/OR” along with addition of synonyms for the keywords. The final search strategy was: ((“CAD/CAM” OR “CAD CAM” OR “Computer assisted design”) AND (“surgical guides” OR “guides” OR “surgical template”) AND “Implant”).

-Study Selection.

Three independent reviewers systematically and independently assessed titles and abstracts of the identified articles following the eligibility criteria outlined below. All three reviewers were able to reach a consensus for all articles accepted for inclusion.

-Eligibility Criteria:

For article inclusion, the following criteria were applied.

**Figure 1:**
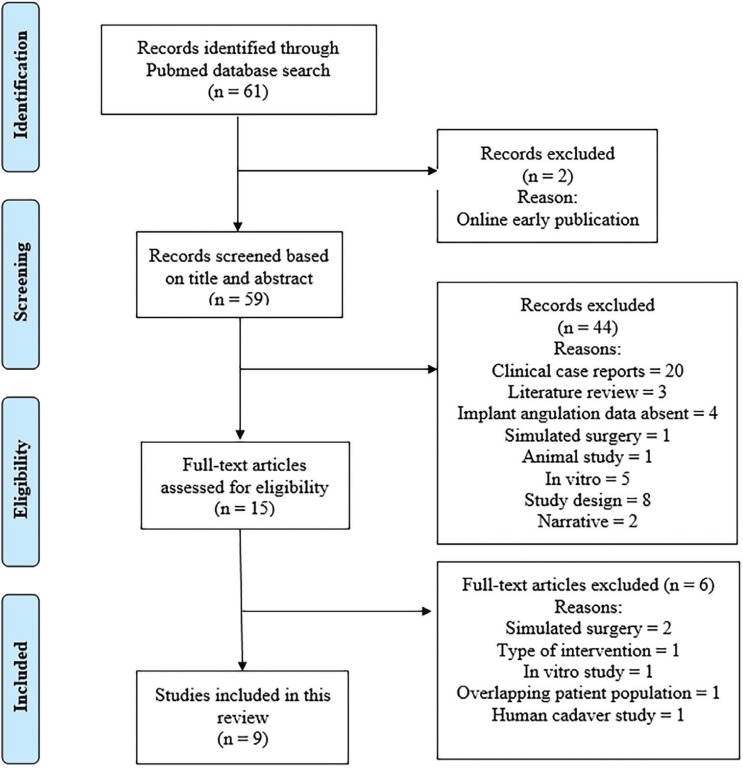
The selection process of the included literature.

1. Study design: clinical studies.

2. Inclusion criteria: Articles published in English within the past 10 years (April 2009-April 2019), studies including placement of 10 implants or more and articles presenting angular deviations in degrees and linear deviations in millimeter (mm).

3. Exclusion criteria: Observational studies, reviews, animal studies, *in vitro*, simulation studies, case reports, narratives and online early published articles. Studies with less than 10 implants placed and studies which did not include data regarding angular deviations in degrees and linear deviations in millimeter (mm) of implants placed. Figure 1 shows the selection process of the included literature in detail.

## Results

-Study selection

A total of 61 potentially relevant titles and abstracts were identified by the electronic search (Medline, Pubmed). After removing 2 online early publications, 59 publications were evaluated based on the title and abstracts. Then, 44 studies were excluded based on abstract evaluation. Fifteen full-text articles were comprehensively examined. At this stage, a total of 6 articles were excluded as they did not meet the inclusion criteria of the present review due to overlapping patient population (36), type of intervention (37) simulated surgery design (38,39), *in vitro* study (25) and human cadaver study (40). The following data were extracted from the 9 eligible studies (22-24,26-29,34,35) that were accepted for inclusion: authors, year of publication, study design, type of CAD/CAM guide utilized, number and types of implants placed, angular deviations in degrees, and linear deviations at implant neck and implant apex in millimeter (mm). Table 1 depicts the detailed information of those publications.

**Table 1:**
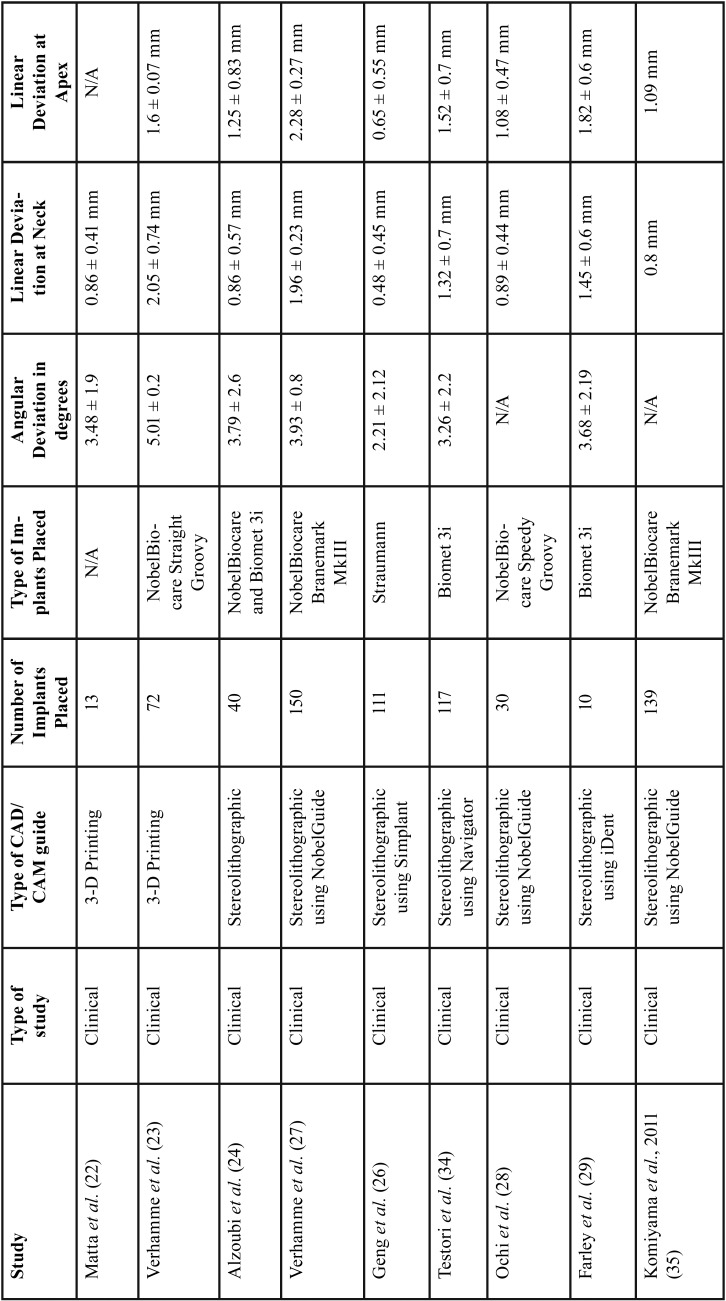
Summary table of the studies included in this review.

-Risk of Bias across studies and quality of evidence:

The quality of the included studies was evaluated by the same three researchers, using the “Risk of Bias In Non-randomized Studies- of Interventions” (ROBINS-I) tool. Categories were ranked low, moderate, serious, or critical (Table 2). Regarding deviations from intended interventions section, all clinical studies were ranked low risk of bias, since planned treatment was rendered in all patients in the study populations. Five clinical trials used only digital planning and superimposition of planned and placed implants, thus were ranked with low risk of bias in the measurement of outcomes category. Four clinical studies utilized digital evaluation and stone casts during planning and superimposition. Due to the dimensional inaccuracy associated with gypsum models, these studies were ranked with moderate risk of bias in the measurement of outcomes category. Clinical studies presenting all deviation values studied for planned versus placed implants were ranked with low risk of bias in the selection of reported results section. Six of the nine articles presented all three deviations (angular deviation, and linear deviations at implant neck and apex), thus they were ranked with low level risk of bias in the missing data category. Clinical studies with greater than 100 implants placed, were ranked with a low risk of bias, while the trials with 10-99 implants place were ranked with moderate risk of bias in the classification of interventions category. Three articles were classified with moderate risk of bias in the missing data category as they had one missing deviation value. None of the studies included in this review were double-blinded randomized clinical trials, thus no study received low risk of bias in the category for selection bias. If patients were selected in a consecutive manner, those articles received moderate risk of bias in the category for selection bias. If participant selection was conducted in a study-specific manner or retrospectively then they were ranked with a serious risk of bias.

**Table 2:**
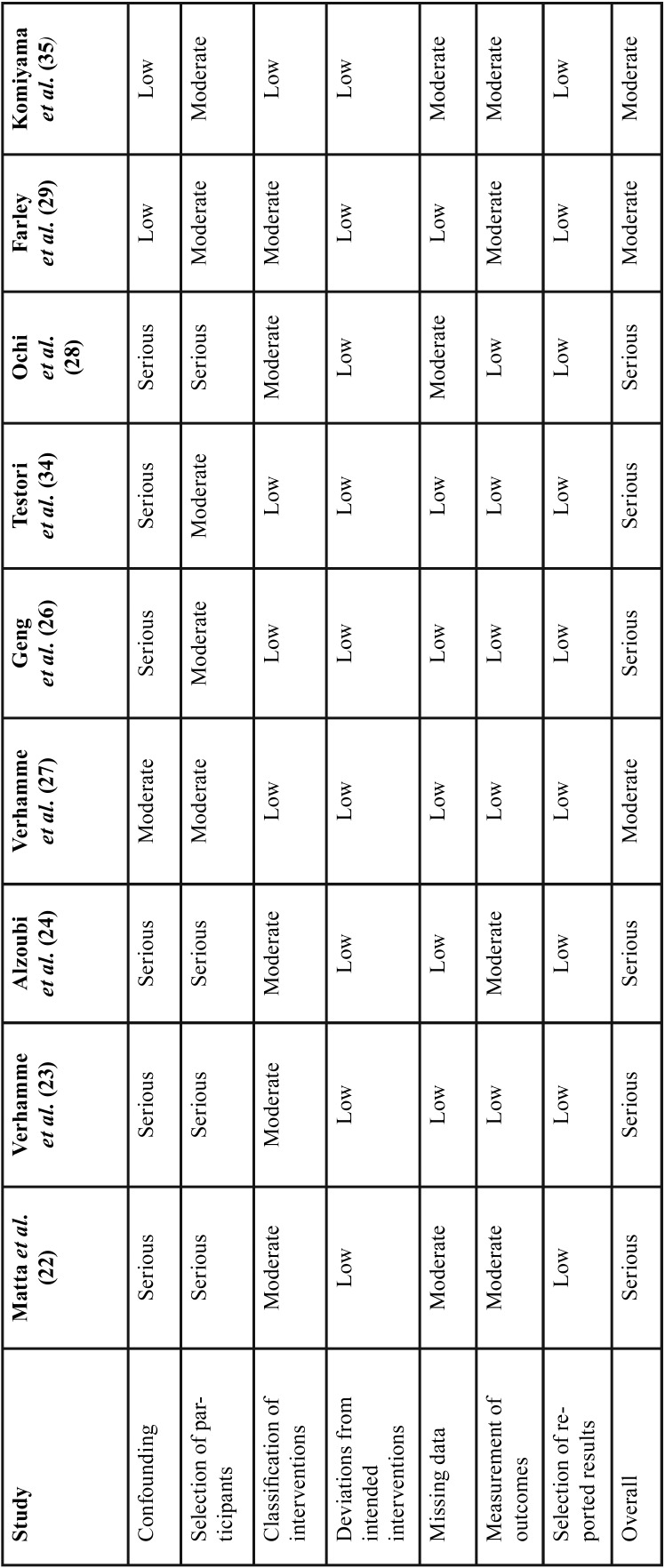
ROBINS-I (risk of bias judgements in non-randomized studies of interventions).

Bone grafting procedures, varying implant locations, unspecified implant locations, multiple implants placed per surgical guides and severe bone resorption were factors considered for the risk of bias in the confounding category. Articles received serious risk of bias if they included the factors mentioned above, while the ones without those factors were ranked with low risk of bias in the confounding category. No discrepancy occurred among the three researchers.

Evaluation of deviations 

**Figure 2:**
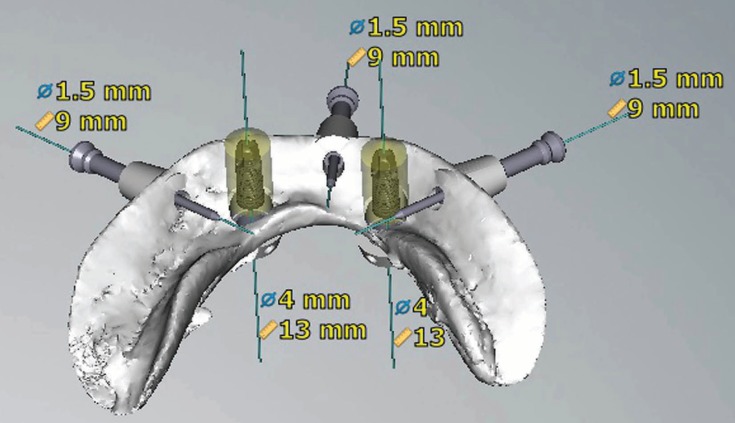
Internal view of the finalized design of a mandibular CAD/CAM surgical guide.

**Figure 3:**
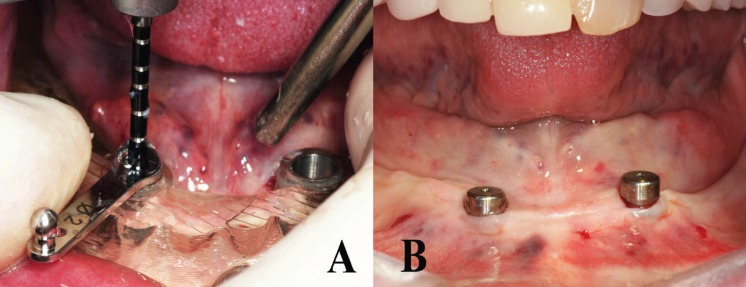
Drilling with a 2mm-diameter drill guide after securing the surgical guide in the mouth (A). Intraoral view of the patient immediately after implant placement using flapless surgical approach (B).

In the nine included studies, various types of software (NobelProcera, Simplant, ident, Stendcad, Mimics) were used to virtually plan implants and design CAD/CAM surgical guides (Figs. 2,3).

When 9 clinical studies were evaluated (22-24,26-29,34,35), a total of 682 implants were inserted. The minimum mean angular deviation, and the linear deviations at implant neck and apex were 2.21 ± 2.12 °, 0.48 ± 0.45 mm, and 0.65 ± 0.55 mm. The maximum mean angular deviation, and the linear deviations at implant neck and apex were 5.01 ± 0.2 °, 2.05 ± 0.74 mm, and 2.28 ± 0.27 mm respectively.

The following common surgical and prosthetic problems were listed in the 9 articles included in this review; poor 3-dimensional virtual implant planning, ill-fitting or broken stereolithographic surgical guides, alterations to the surgical plan, early implant loss due to poor primary stability and ill-fitting or broken prosthetic restorations especially immediate temporary ones.

## Discussion

Recently, the implant insertion with CAD/CAM surgical guides has become popular. Despite enthusiastic expectations regarding computer guided implant placement, a few reports identified the risk of errors and complications that clinicians may face from the digital planning to the actual implant surgery (33). In this study, the dental literature was reviewed and the key points from previous studies were discussed. Despite the fantastic belief of absolute precision of CAD/CAM surgical guides, which is not uncommon among novice practitioners, there is no perfect computer-guided implant surgery in real life. Because, several authors reported certain level of deviations from digital planning to the actual implant placement (30-32).

In clinical studies indicated that the mean angular deviation, and the linear deviations at implant neck and apex were up to 5.01 ± 0.2 °, 2.05 ± 0.74 mm, and 2.28 ± 0.27 mm respectively (22-24,26-29,34,35). When compared to *in vitro* studies, the greater errors were reported in the clinical studies. Because, most factors that may contribute errors can be kept under control in laboratory environment but many of them can not be controlled in the clinical situations.

Verhamme *et al.* (27), aimed to determine the clinical accuracy of implant placement using computer planning and CAD/CAM surgical templates. They placed 150 implants in 25 edentulous patients by using flapless surgical technique. A CBCT scan was acquired pre-operatively and post-operatively to determine variation in deviations of planned and placed implants. They observed that the angular deviation and linear deviations at the implant neck and apex were 3.93 ± 0.8°, 1.96 ± 0.23 mm and 2.28 ± 0.27 mm respectively. They concluded that the clinicians should be aware of a certain level of deviations which may stem from angulations and translations of the surgical guide.

Turbush and coworkers (31) analyzed the accuracy of implant placement by using 3 different types of stereolithographic surgical guides (bone-supported, tooth-supported, and mucosa-supported). A total 150 implants were virtually planned in a 3-D software program, and then placed in 30 photopolymer resin mandibles. They compared the virtual implant placement with the actual implant placement by superimposing pre-surgical and post-surgical CBCT scans. The mean angular deviation of the long axis between the planned and placed implants was 2.2 ±1.2 degrees. They also noted that the mean linear deviations between the planned and placed implants were 1.18 ±0.4 mm at the implant neck and 1.44 ±0.6 mm at the implant apex for all 150 implants.

Cushen and coworkers (30) investigated the effect of operator experience on implant placement accuracy with stereolithographic surgical guides. In their study, a total of 100 implants were placed in 20 photopolymer resin mandibles by 4 operators (2 experienced and 2 inexperienced in implant placement). With the help of pre- and post-operative CBCT scans, the amount of angular, horizontal, and vertical deviation of the placed implants from the virtually planned implants at the neck and apex was measured. They observed that the mean angular deviation was 2.6 ±1.2 degrees, the horizontal linear deviations at the platform and the apex were 0.63 ± 0.3 mm and 0.34 ± 0.1 mm for the experienced operators. The corresponding Figures were 3.96 ± 1.6 degrees, and 0.77 ± 0.3 mm and 0.42 ± 0.2 mm for the inexperienced group. They concluded that the experience level of the operator contributes to the accuracy of implant placement, with more experienced operators placing more implants accurately.

It is cardinal to mention here that there is a learning curve for this method like other innovative procedures. Practitioners who want to utilize this advanced procedure should acquire comprehensive training (30,31), otherwise, surgical and restorative complications may occur. Some of those complications that may be caused by untrained and inexperienced operators may be major such as inferior alveolar nerve damage. Today, only about 15% of overall number of dentists place and/or restore implants and only about 1% of the dentists know how to use 3-dimensional implant planning software and hardware. In addition, the number of faculty in dental schools providing trainings regarding CAD/CAM surgical guides is very limited, which makes obtaining impeccable training about this advanced technique harder for the novice operators.

Generally, clinical studies present greater deviations between planned and actual placed implants. Because, there are many critical clinical steps from inserting the CAD/CAM surgical guide to the complete seating of the implants, and factors such as limited mouth opening, difficulty in securing the surgical guide with anchors pins that may compromise the overall precision. In a clinical study, Verhamme and coworkers (23) placed 72 implants in 12 edentulous maxillae by means of a certain type of stereolithographic surgical guide (Nobelguide, NobelBiocare, CA, USA). They observed that the mean angular deviation was 5.02 ± 0.19 °. They also noted that the mean deviations at the implant shoulder and the implant tip were 2.05 ± 0.07 mm and 1.59 ± 0.07 mm in the three dimensional direction.

Alzoubi and coworkers (24) aimed to compare the accuracy of 40 implants placed in 29 patients by using immediate (25 implants) and delayed (15 implants) placement protocols. All implants were placed with CAD/CAM surgical guides after planning with a certain type of 3-dimensional software (Anatomage Invivo5, Anatomage, San Jose, CA, USA). They found that the overall mean angular deviation were 3.8 degrees and the linear deviations at the implant platform and apex were 0.86 mm and 1.25 mm respectively.

## Conclusions

It has been suggested that despite implant placement using CAD/CAM surgical guides has recently become a popular method, there is no perfect accuracy in the clinical situation. This review indicated that the operators should be aware of the angular and linear deviations up to 5 ° and 2.3 mm. Hence, it vital to emphasize that novice dentists should obtain comprehensive training and supervision before they place implants with CAD/CAM surgical guides to avoid serious complications. Lastly, the operators need to establish a safety zone between implants and critical anatomic structures such as inferior alveolar nerve during selecting the location and length of the implants.
